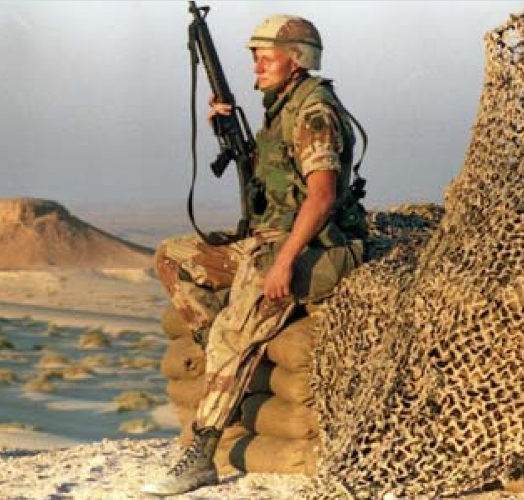# The Beat

**Published:** 2008-05

**Authors:** Erin E. Dooley

## Navajo Fight Uranium Comeback

In 2005 the Navajo nation banned uranium mining and milling on its lands because of increases in lung cancer and other chronic diseases in miners and residents living near piles of mine waste. Now, because of soaring uranium prices and the challenge to find cleaner domestic sources of energy, mining companies are seeking state permits to mine the estimated 500 million pounds of uranium reserves located in and near the Navajo lands. In late 2007 the Navajo nation asked Congress for a moratorium on uranium mining on Indian lands despite pending state legislation that would require mining companies to set aside a small percentage of their profits as a “legacy fund” to clean up existing contaminated sites and the promise from the companies to use more sustainable mining methods.

**Figure:**
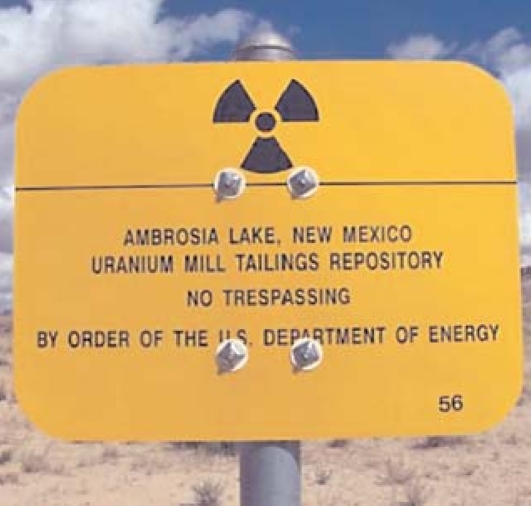


## New Lead Paint Rule

On 31 March 2008, the EPA issued long-awaited new regulations on reducing children’s exposure to lead paint during renovations and repairs of buildings constructed before 1978. The regulations will go into effect in April 2010, at which time contractors and maintenance professionals working in pre-1978 housing, child-care facilities, and schools must be trained and certified in lead-safe practices. The EPA is initiating an education and outreach campaign to alert the industry to these new requirements. Despite industry estimates that the new rules will drive up renovation costs, several senators have announced their plans to introduce more stringent legislation that would reduce loopholes in the current rules and require more testing to ensure vulnerable populations are thoroughly protected.

## Race Without a Trace

In support of the ongoing international trend toward making sporting events more environmentally friendly, the North Carolina-based *Endurance Magazine* has launched the “Race Without a Trace” initiative to minimize the waste and carbon emissions generated by endurance events. The initiative focuses on producing events with a carbon-neutral strategy by purchasing carbon offsets, reducing the amounts of paper used for marketing and distribution at races, encouraging the use of eco-friendly portable toilets at events, and composting leftover food and paper. The next level of effort includes finding alternative energy sources for finish-line electronics and minimizing the shipping required for race day materials and goods.

**Figure:**
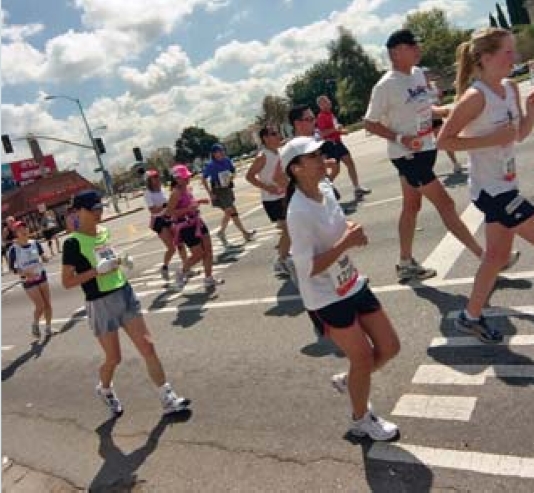


## Nanofood on Research Menu

For the past few years, the food industry has spent millions of dollars on nanotechnology research and development, but there has been little government oversight concerning the safety and regulation of “nanofoods” (foods produced with or packaged using nanomaterials). The authors of a report in the March 2008 issue of *Food Additives and Contaminants* say the uncertainties posed by the lack of data on the health effects of nanofoods warrant action at the regulatory and research levels, as large segments of the population are likely to consume such products without knowing it. *Out of the Laboratory and on to Our Plates*, an April 2008 report by Friends of the Earth, further recommends that ”nano-” be redefined to include particles up to 300 nm in size, which may also pose health risks.

**Figure:**
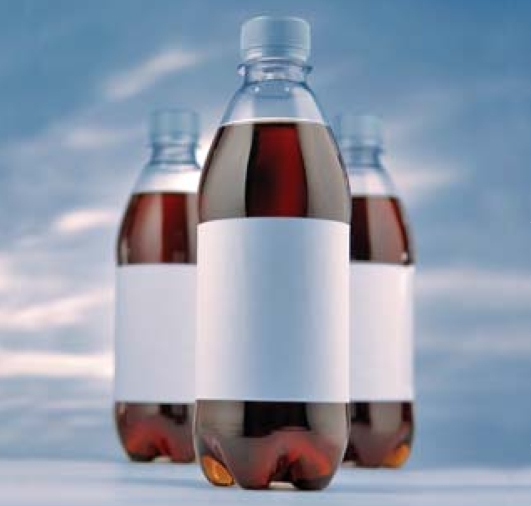


## Key to Reducing CO_2_?

Each year, buildings release 35% of the total CO_2_ emitted in North America. In March 2008, the trinational Commission for Environmental Cooperation released *Green Building in North America: Opportunities and Challenges*, which prescribes green design, construction, renovation, and operation as ways to reduce emissions more significantly, cheaply, and quickly than other available means. Among the report’s recommendations are that the North American governments establish joint green building guidelines and bolster research and development efforts in this area.

## New Findings on Gulf War Illness

Increasing evidence suggests that chronic multisymptom illness in Persian Gulf War veterans can be partly explained by exposure to organophosphate and carbamate acetyl-cholinesterase inhibitors (AChEis) in the nerve agent sarin, anti–nerve gas pills, and pesticides used to control vectorborne diseases. A review published in the 18 March 2008 issue of the *Proceedings of the National Academy of Sciences* demonstrates that such exposures may be linked to multisymptom health problems in 25–33% of returning Gulf War veterans. The author also presents evidence that genetics may play a role in the illness—some individuals have lower activity of enzymes that detoxify AChEis, thereby intensifying their susceptibility to the chemicals’ effects.

**Figure:**